# Striatonigral neurons divide into two distinct morphological-physiological phenotypes after chronic L-DOPA treatment in parkinsonian rats

**DOI:** 10.1038/s41598-018-28273-5

**Published:** 2018-07-03

**Authors:** T. Fieblinger, L. Zanetti, I. Sebastianutto, L. S. Breger, L. Quintino, M. Lockowandt, C. Lundberg, M. A. Cenci

**Affiliations:** 1 Basal Ganglia Pathophysiology Unit, Department of Experimental Medical Science, Lund University, Lund, Sweden; 2 CNS Gene Therapy, Department of Experimental Medical Science, Lund University, Lund, Sweden; 3 Wissenschaftskolleg zu Berlin, Institute for Advanced Study, Wallotstr. 19, D-14193 Berlin, Germany; 4 Institute of Pharmacy, Pharmacology and Toxicology, Center for Molecular Biosciences, University of Innsbruck, Innsbruck, Austria; 5 CNRS, Institut des Maladies Neurodégénératives, University of Bordeaux, Bordeaux, France

## Abstract

Dendritic regression of striatal spiny projection neurons (SPNs) is a pathological hallmark of Parkinson’s disease (PD). Here we investigate how chronic dopamine denervation and dopamine replacement with L-DOPA affect the morphology and physiology of direct pathway SPNs (dSPNS) in the rat striatum. We used a lentiviral vector optimized for retrograde labeling (FuG-B-GFP) to identify dSPNs in rats with 6-hydroxydopamine (6-OHDA) lesions. Changes in morphology and physiology of dSPNs were assessed through a combination of patch-clamp recordings and two photon microscopy. The 6-OHDA lesion caused a significant reduction in dSPN dendritic complexity. Following chronic L-DOPA treatment, dSPNs segregated into two equal-sized clusters. One group (here called “cluster-1”), showed sustained dendritic atrophy and a partially normalized electrophysiological phenotype. The other one (“cluster-2”) exhibited dendritic regrowth and a strong reduction of intrinsic excitability. Interestingly, FosB/∆FosB induction by L-DOPA treatment occurred preferentially in cluster-2 dSPNs. Our study demonstrates the feasibility of retrograde FuG-B-GFP labeling to study dSPNs in the rat and reveals, for the first time, that a subgroup of dSPNs shows dendritic sprouting in response to chronic L-DOPA treatment. Investigating the mechanisms and significance of this response will greatly improve our understanding of the adaptations induced by dopamine replacement therapy in PD.

## Introduction

Spiny projection neurons (SPNs) are classically divided into two major subpopulations based on anatomical and functional properties^1–3^. Anatomically, direct-pathway SPNs (dSPNs) project to the substantia nigra pars reticulata (SNr), and indirect-pathway SPNs (iSPNs) send their axons to the globus pallidus pars externa (GPe). Functionally, dSPNs and iSPNs are distinguished by their preferential expression of dopamine (DA) receptor D1 and D2, respectively. Direct- and indirect pathway SPNs are also assigned antagonistic roles in the control of behavior. Activation of the direct pathway is considered to promote action selection and movement initiation, whereas activation of the indirect pathway suppresses action selection and inhibits movement, even though multiple lines of research cast doubt on such a clear-cut segregation^4,5^. Cellular morphology, and especially the extent of dendritic arborization, impacts on the intrinsic excitability of SPNs. Thus, dSPNs show larger arborizations and are less excitable than iSPNs in healthy mice^6^. This difference in excitability has also been observed in rat SPNs^7^. Interestingly, experimentally inducing hyper-excitability in SPNs causes marked dendritic regression^8^, arguing for causal morphology-physiology relationship.

Dendritic atrophy or malformation of SPNs occurs in several basal ganglia disorders and it is likely to impact on these neurons’ synaptic connectivity and intrinsic excitability^9^. Human post-mortem studies of the striatum in Parkinson’s disease (PD) reported severe shrinkage of SPN dendrites^10–12^. They could however not specify whether both SPN populations were equally affected, nor whether the dendritic atrophy was caused by the primary disease or by DA replacement therapy, which leads to the development of L-DOPA-induced dyskinesia (LID) in the majority of PD patients. We and others have addressed these questions using mouse models of PD^3,13,14^. Although PD has a complex pathology, striatal DA denervation by means of 6-OHDA lesions was sufficient to induce dendritic atrophy in both SPN populations and a short course of L-DOPA treatment did not reverse this effect^3^. Our results regarding dendritic atrophy in parkinsonian mice were confirmed in one^13^, but not another study^14^. Furthermore, a study applying the Golgi-Cox silver impregnation technique to rats with 6-OHDA lesions failed to detect dendritic atrophy in unidentified SPNs^15^.

In the present study, we set out to examine the morphological-physiological properties of identified dSPNs in a chronic rodent model of PD exposed to prolonged L-DOPA treatment. To this end, we chose to use rats instead of mice because rats exhibit a stable behavioral and molecular phenotype over many months after complete nigrostriatal lesions (reviewed in^16^). In order to unequivocally identify dSPNs we utilize a novel retrograde labeling approach. We focused on dSPNs because an unequivocal identification of iSPNs is not achievable with retrograde labeling methods, as the main projection target of the iSPNs (the GPe) also receives a substantial amount of bridging collaterals from striatonigral neurons. Retrogradely labeled FuG-B-synapsin-GFP (FuG-B-GFP)-positive dSPNs showed clear dendritic atrophy after dopamine denervation, as well as electrophysiological alterations that closely resemble those reported from 6-OHDA lesioned mice^3^. Surprisingly, though, L-DOPA treatment elicited a complex response segregating the dSPNs into two different phenotypes: half of the neurons maintained the same levels of dendritic regression as produced by the lesion, whereas the other half showed increased dendritic arborization, associated with a strong reduction in excitability. Our data further suggest that the dendritic sprouting of some SPNs during chronic L-DOPA treatment may depend on a high expression of ∆FosB.

## Results

### Labeling of direct pathway striatal neurons using FuG-B-GFP viral vectors

**Figure 1:**
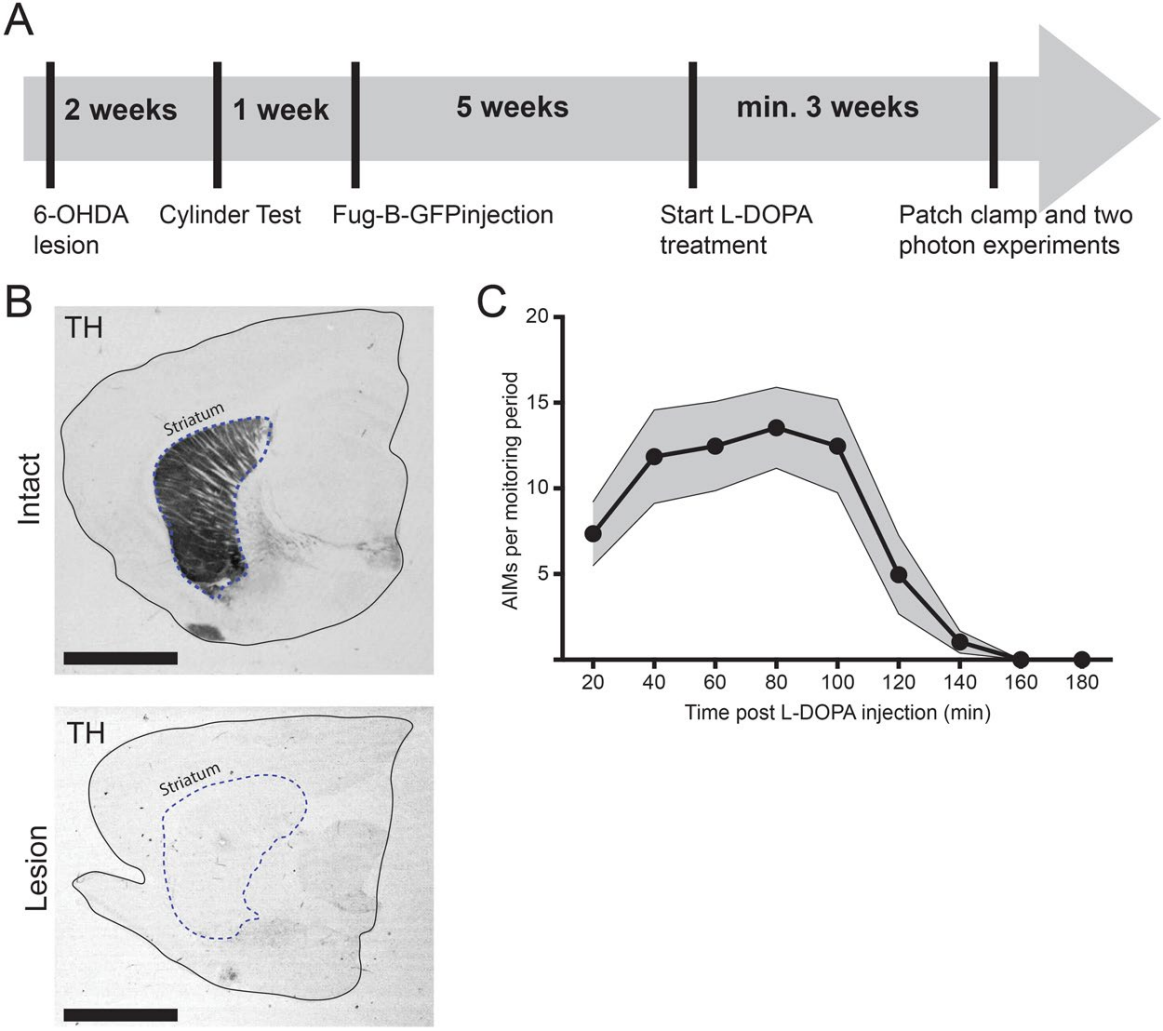
Experimental outline. (**A**) Time course of the experiment. Rats were injected with 6-OHDA and screened for forelimb use asymmetry two weeks later. After one additional week, FuG-B-GFP viral vector was injected into the substantia nigra pars reticulata, and *ex vivo* experiments started earliest eight weeks after. Daily L-DOPA treatment started six weeks after 6-OHDA lesion and lasted 3–4 weeks. (**B**) Representative tyrosine hydroxylase staining (TH). Near-complete elimination of TH in the striatum in the hemisphere ipsilateral to the toxin injection was documented for all animals in this study. Scale bars: 0.5 cm (**C**) Representative plot of AIM scores post L-DOPA injection for a testing session after three weeks of treatment (n = 7).

**Figure 2:**
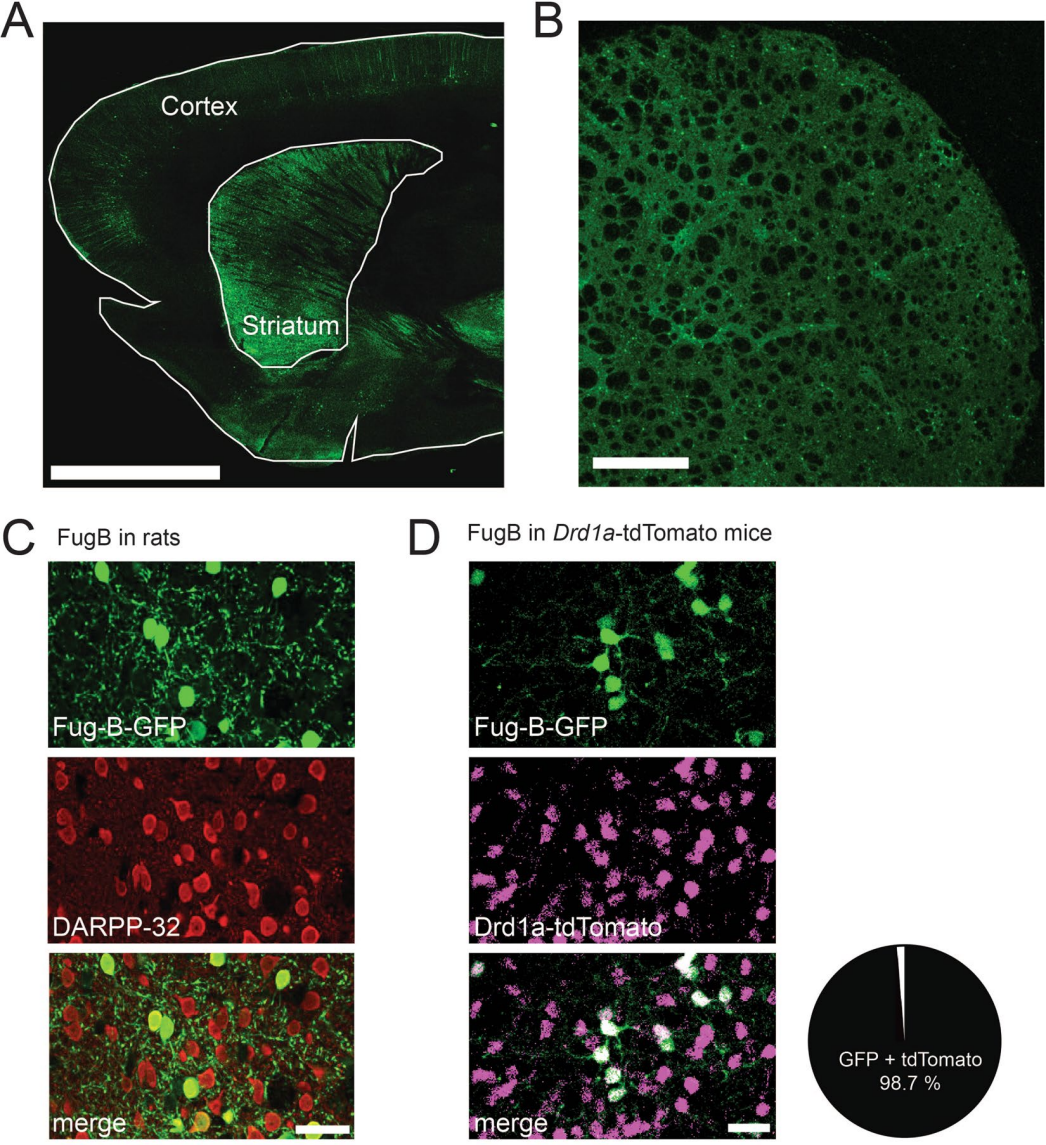
FuG-B lentiviral labelling of ‘classical’ dSPNs in rat and mouse. (**A**) Overview image of a sagittal rat brain section. GFP-positive cells are found throughout the striatum, but to lesser extent also in the cortex. Scale bar: 0.5 cm (**B**) Low magnification confocal image of a coronal brain section shows FuG-B-GFP-labeled cells throughout striatum. Scale bar: 500 μm. (**C**) Co-labeling shows that these FuG-B-GFP positive cells in the rat striatum are positive for DARPP-32, a marker of SPNs. Scale bar: 25 μm. (**D**) We found 98.7% of co-labelling of FuG-B-GFP and strong tdTomato fluorescence after injecting the same vector in transgenic BAC-*drd1a*-tdTomato mice (n = 152 cells in 6 mice). Scale bars: 25 μm.

In order to identify striatal SPNs that directly project to the SNr, we injected the FuG-B-GFP lentiviral vector in the rat SNr for retrograde labeling^17^. After 8 weeks (see Fig. 1 for experimental outline) we found robust expression of GFP in the striatum (Fig. 2A,B). Labeled neurons had the typical appearance of striatal SPNs and co-localized with the SPN marker dopamine- and cAMP-regulated neuronal phosphoprotein, 32 kDa (DARPP-32) (Fig. 2C). To show that FuG-B-GFP labels striatonigral neurons with a ‘classical’ dSPN phenotype, we injected the same construct into the SNr of transgenic mice that express a red fluorophore under the dopamine D1-receptor promoter (BAC-*drd1a*-tdTomato mice). In these mice, virtually all striatal cells labeled with FuG-B-GFP also co-expressed tdTomato (Fig. 2C). These results validate the FuG-B-GFP viral vector as a suitable tool to retrogradely label ‘classical’ D1-receptor-positive dSPNs.

### Dendritic atrophy in the rat model of Parkinson’s disease is reversed in a subgroup of dSPNs by chronic L-DOPA treatment

**Figure 3:**
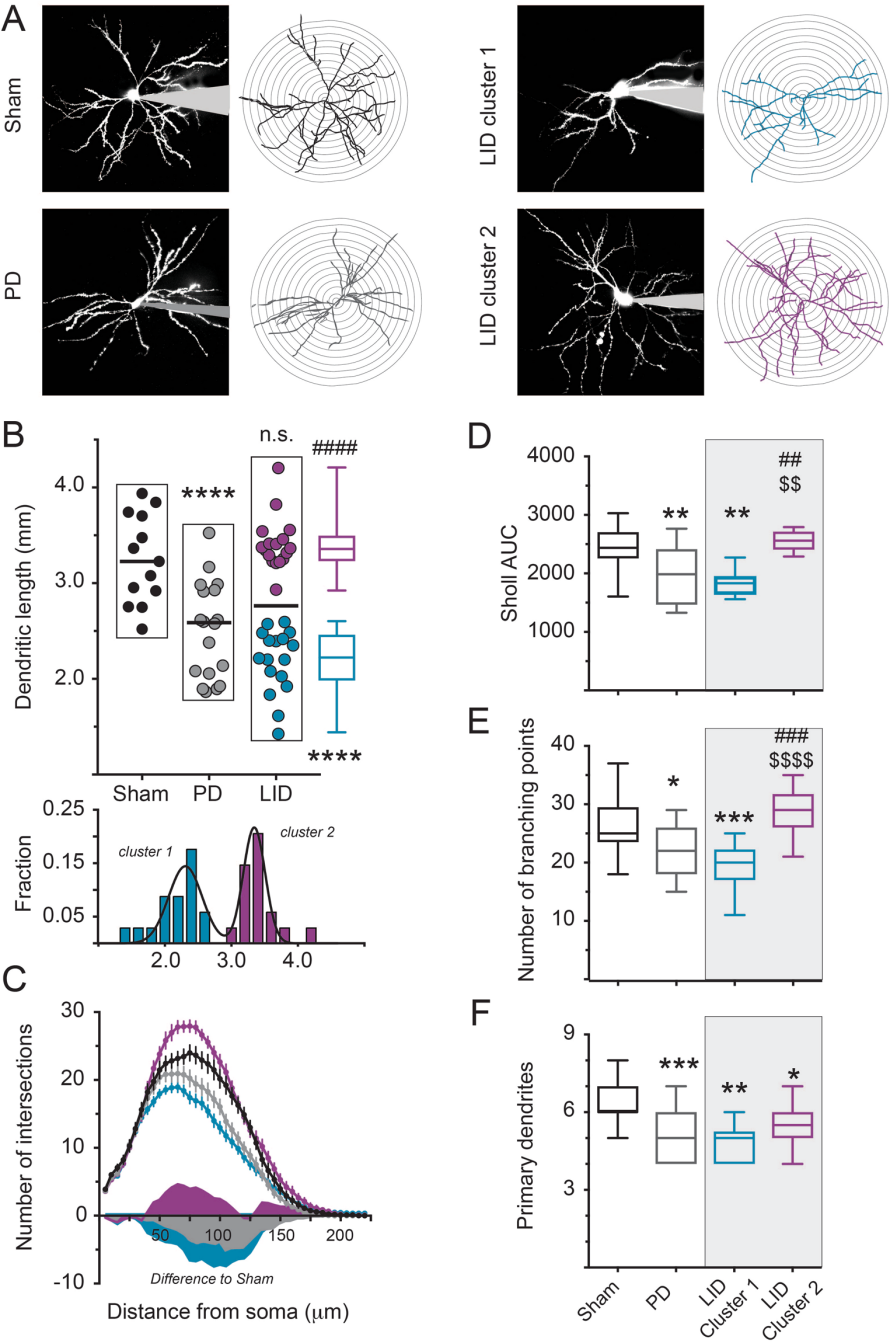
Dendritic reconstructions reveal two morphologically distinct clusters of dSPNs in dyskinetic rats. (**A**) Examples of 3D reconstructed neurons. Maximum intensity projections of patch-filled dSPNs are shown paired with the corresponding reconstructions. (**B**) After L-DOPA treatment, dSPNs segregate into two cluster. Total dendritic length is significantly decreased in experimental parkinsonism (n = 13 and 18). However, pooled dSPNs in LID were not significantly different from neither sham nor PD group (n = 34). Closer inspection reveals that these neurons split into two clusters (each n = 17). Similar to neurons in the PD group (*gray*), cluster-1 dSPNs (*blue*) display significant atrophy. However, cluster-2 dSPNs (*magenta*) show significantly increased dendritic length compared to the PD group and are not different from control cells (*black*). The histogram of the LID group dSPNs shows that the distribution is indeed best fit as a sum of two gaussian functions, with two separate peaks. (**C**) Sholl analysis shows a loss of dendritic intersections at various distances from the soma for both PD group and cluster-1. Cluster-2 dSPNs, however, displays even more intersections than control cells in the mid-distance from the soma. (**D**) The area under the curve (AUC), as calculated from the Sholl analysis, confirms dendritic atrophy in cells of the PD group and LID cluster-1 dSPNs. Conversely, cluster-2 dSPNs are not significantly different from neurons in the sham group. (**E**) While the number of dendritic branching points is reduced in both PD group and cluster-1 dSPNs, it is indistinguishable from sham in cluster-2 dSPNs. (**F**) The number of primary dendrites was reduced in all cells as compared to sham. *^,^**^,^***p < 0.05/0.01/0.001 vs. sham; ^##,###,####^p < 0.01/0.001/0;0001 vs. PD; ^$$,$$$$^ p < 0.01/0.0001 vs. cluster-1 (Tukey’s multiple comparison test).

We prepared acute slice preparations to investigate the dendritic arborization of FuG-B-GFP-labelled dSPNs in the dorsolateral striatum following chronic treatment with L-DOPA or vehicle (see Fig. 1). To visualize the entire dendritic arbour, we filled GFP-labelled cells with Alexa 568 via a patch-pipette and imaged them using two photon laser scanning microscopy (2PLSM). In agreement with findings from the human PD striatum^10–12^ and mouse models of PD^3^, dSPNs displayed marked dendritic atrophy with a drastically reduced dendritic length (Fig. 3A,B).

Three weeks of treatment with L-DOPA (10 mg/kg/day) induced moderate-severe dyskinesia in all rats included in this study (Fig. 1C). Reconstructed dSPNs from dyskinetic animals (LID group) showed a wide-spectrum of dendritic lengths that, taken together, did not differ significantly from the values measured in either sham-lesioned controls (sham group) or 6-OHDA-lesioned rats treated with vehicle (PD group) (Fig. 3A,B). However, upon close examination, data from the LID group appeared to separate into two clusters. Cluster-1 dSPNs showed a reduced dendritic length, with values significantly lower than for the sham cells and indistinguishable from those in the PD group. Cluster-2 dSPNs had, on the contrary, an increased dendritic length as compared to the PD group, and were not significantly different from the sham cells (Fig. 3B). Confirming this observation, the histogram of dendritic lengths in the LID group was best fitted as the sum of two Gaussians (R^2^ = 0.89), indicating the existence of two separate subpopulations (Fig. 3B *bottom*). This separation was apparent also in other morphological parameters. Scholl analysis revealed a reduced dendritic arborization in the PD group, which was maintained in cluster-1 dSPNs, but reversed in cluster-2 dSPNs after L-DOPA treatment (Fig. 3C,D). The number of branching points was reduced in both the PD group and cluster-1 dSPNs yet recovered to sham control-levels in cluster-2 dSPNs (Fig. 3E). Interestingly, the loss of primary dendrites in the PD group was not recovered in either of the LID cell clusters, indicating that the overall recovery of dendritic arborization must have stemmed from increased branching of existing dendrites, rather than from the re-growing of lost dendrites (Fig. 3F). It is important to note that dyskinetic rats never harbored only one or the other cluster of dSPNs, but that both types of cells could be found within any given animal. There was furthermore no apparent spatial segregation of the two clusters in the dorsolateral striatum. Following the observation that L-DOPA triggers two types of morphological responses in dSPNs, we investigated if this entails physiological differences as well.

### Morphological phenotypes concur with electrophysiological differences in the two dSPN cluster

It has been proposed that dendritic morphology is a crucial determinant of somatic excitability in SPNs^6,8^, even though other factors can also shift the SPN excitability to higher or lower levels under pathophysiological conditions^3,18^. We and others have reported increased excitability of dSPNs in 6-OHDA-lesioned mice, as indicated by more action potential firing upon somatic current injections^3,14,19–21^.

**Figure 4:**
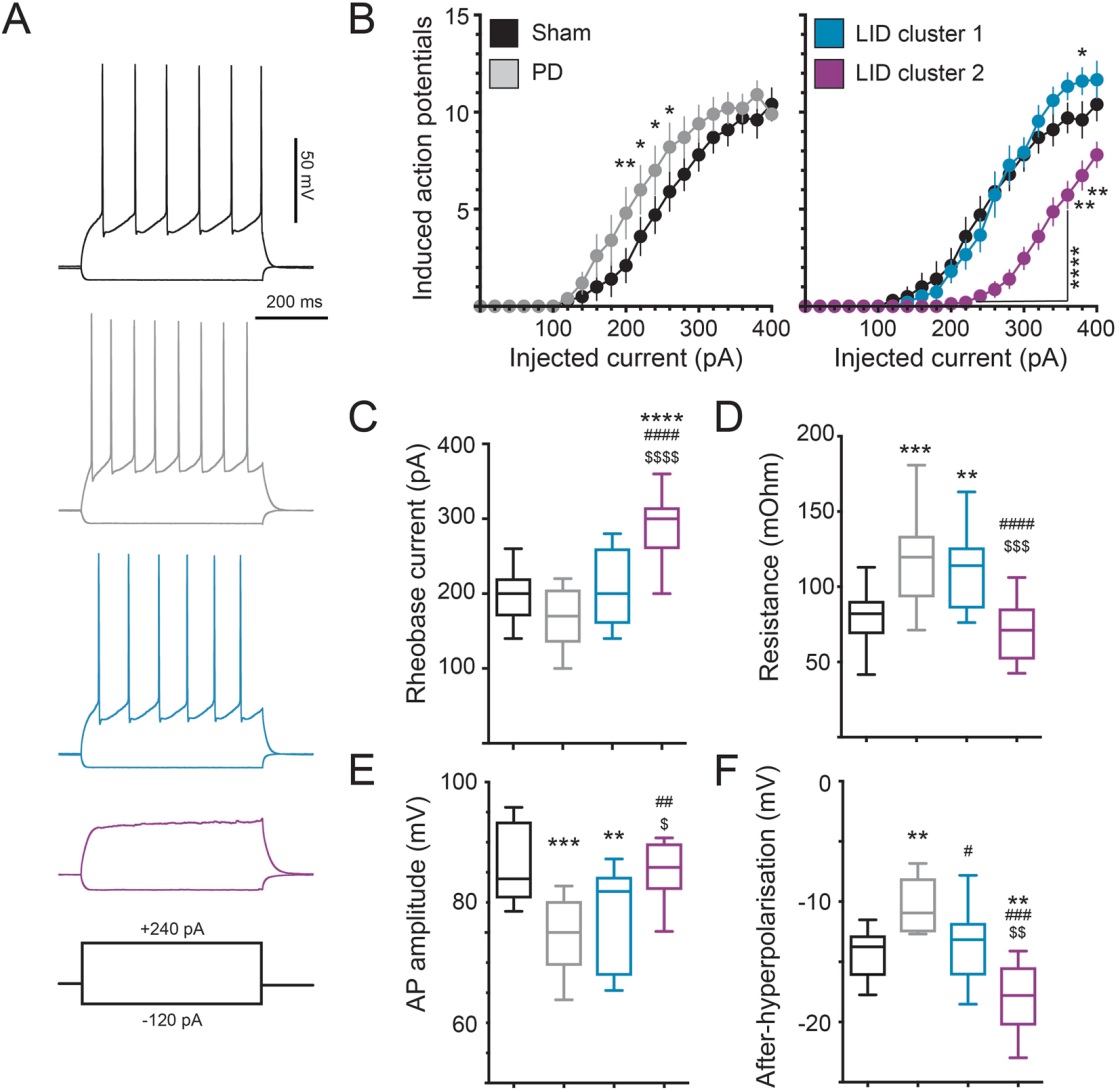
Electrophysiological properties confirm the separation into two subpopulations of dSPNs in LID. (**A**) Example recordings for all groups of neurons are shown. Each trace depicts the voltage change in response to a hyper- (−120 pA) and a depolarizing (240 pA) current injection. Note that this depolarizing current is sufficient to induce action potential firing in sham, PD and cluster-1 dSPNs, however not in cluster-2 dSPNs. (**B**) Plotting the evoked action potentials over the injected current shows a slight left shift in the PD curve towards a more excitable state (*left diagram*, n = 13 and 18). The curve for cluster-1 dSPNs is nearly indistinguishable from the one of sham neurons (*right diagram*, n = 17). However, cluster-2 dSPNs showed a clear decrease in excitability and a shift to the right (n = 17). (**C**) This dramatic shift in excitability of cluster-2 dSPNs is also represented in the significant increase of the rheobase current. (**D**) Input resistance is increased in the same way for both, neurons in the PD group and cluster-1 dSPNs. However, input resistance decreased to sham levels in cluster-2 dSPNs. (**E**) Action potential amplitude also dropped in the cells from PD rats and was not altered in cluster-1 dSPNs. Values for cluster-2 dSPNs were however increased and not different from sham levels. (**F**) The after-hyperpolarization decreased with DA-denervation and was partially restored in cluster-1 dSPNs. Cluster-2 dSPNs showed a clearly stronger after-hypoerpolarizion than cluster-1 dSPNs, PD and even sham group. *^,^**^,^***^,^****p < 0.05/0.01/0.001/0.0001 vs. sham, ^##,###,####^p < 0.01/0.001/0.0001 vs. PD, ^$,$$,$$$,$$$$^p < 0.05/0.01/0.001/0.0001 vs. cluster-1 dSPNs (Tukey’s multiple comparison test).

Using this rat model we could now establish that dSPNs show an increased excitability even after more than eleven weeks post lesion (Fig. 4A,B), albeit not as pronounced as that found in the analogous mouse model^3^. Surprisingly, L-DOPA treatment had different effects on the two morphologically distinct dSPN clusters. Cluster-1 dSPNs, with sustained dendritic atrophy, showed a normalization of intrinsic excitability back to sham-control levels. In contrast, cluster-2 dSPNs (the cells with increased dendritic arborization) showed a markedly reduced excitability, with significantly less action potential firing than sham-control cells (Fig. 4A,B). Moreover, the rheobase current (i.e. the minimal somatically injected current to elicit action potentials) was significantly increased in cluster-2 dSPNs as compared to all the other groups (Fig. 4C).

In line with our previous mouse data^3^, we found that several passive parameters were altered in the parkinsonian state, with dSPNs having a higher input resistance, lower action potential amplitude and reduced after-hyperpolarization (AHP) compared to the sham group (Fig. 4D–F). Interestingly, none of these parameters was affected by L-DOPA treatment in cluster-1 dSPNs, with the exception of the AHP, which was restored to sham control levels. Conversely, L-DOPA had a strong effect in cluster-2 dSPNs, reversing the lesion-induced changes in input resistance, action potential amplitude, and amplitude of AHP. These changes may partially explain the decreased excitability of cluster-2 dSPNs.

Taken together, our data show that dSPNs in the parkinsonian rat brain show two distinct responses to L-DOPA treatment. Cluster-1 dSPNs maintain the denervation-induced dendritic atrophy although their excitability is restored to sham-control levels. On the other hand, cluster-2 dSPNs show a remarkable increase in dendritic arborization that is paralleled by a pronounced reduction in excitability below the levels measured in non-lesioned animals.

### L-DOPA-induced FosB/∆FosB expression occurs in cluster-2 dSPNs

Having observed two distinct morphological and electrophysiological phenotypes in dSPNs after prolonged L-DOPA treatment, we set out to explore the underlying molecular changes. One well-described molecular mediator of LID development is transcription factor FosB and its truncated splice variant ∆FosB, which are induced by L-DOPA in dSPNs^22,23^.

**Figure 5:**
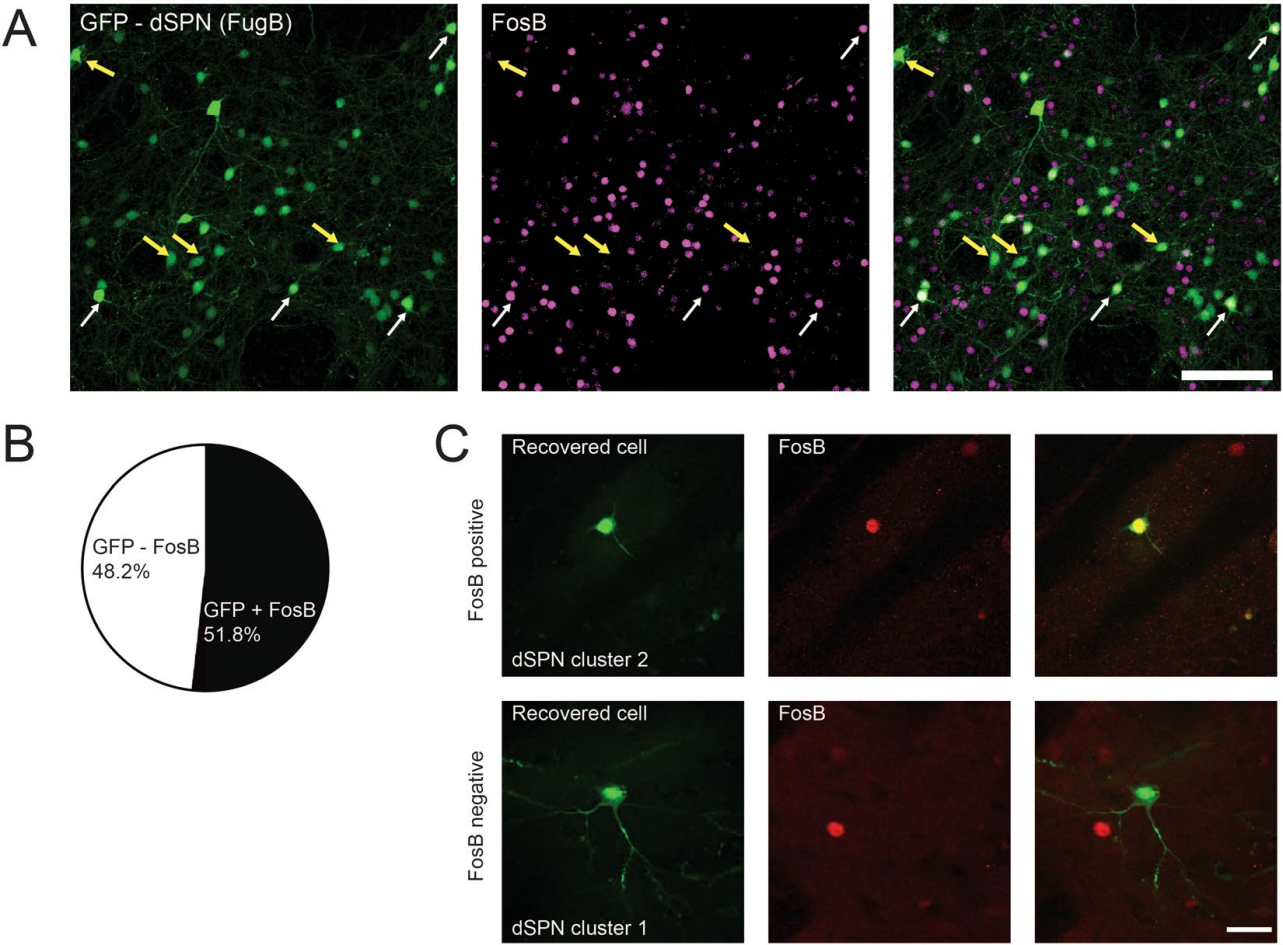
FosB/∆FosB expression in FuG-B-GFP labeled SPNs. (**A**) Striatal sections containing FuG-B-GFP labeled cells (*green*) were co-stained for FosB/∆FosB (*magenta*). Approximately half of the GFP-positive cells showed clear staining for FosB/∆FosB (*white arrows*), whereas the other half did not (*yellow arrows*). Scale bar: 100 μm. (**B**) Analysis of n = 873 GFP neurons from 5 rats established that 51.8% of the retrogradely labeled dSPNs also expressed FosB-like immunoreactivity. (**C**) Some cells that were used for patch clamp and 2PLSM experiments were recovered in fixed slices based on their expression of an Alexa dye. Slices were sectioned and immunostained for FosB/∆FosB. The examples show a cluster-2 dSPN with clear nuclear immunostaining (*upper row*) and a cluster-1 dSPN that is clearly FosB-negative (*lower row*). Scale bar: 25 μm.

Sections from FuG-B-GFP-injected animals chronically treated with L-DOPA were immunostained with an antibody recognizing FosB and ∆FosB (FosB/∆FosB). Matching our observation that dSPNs separate into approximately two equal-sized subpopulations, we also found that about half of the FuG-B-GFP-positive cells showed clear nuclear staining for this transcription factor (Fig. 5A,B). To test whether FosB/∆FosB activation occurs selectively in cells of either cluster-1 or 2, we attempted to recover dSPNs after electrophysiological recording and 2PLSM imaging and were able to recover a total of 11 cells. FosB/∆FosB staining revealed that 100% of the recovered cells with positive immunoreactivity were cluster-2 dSPNs, whereas 87.5% of the FosB-negative recovered dSPNs belonged to cluster-1 (Fig. 5C). Our data therefore suggest that FosB/∆FosB could be driving the differentiation of dSPNs into two distinct phenotypes after L-DOPA treatment.

## Discussion

Our study provides two important new findings. First, we demonstrate the feasibility of a retrograde labeling with FuG-B-GFP to study dSPNs in the rat striatum. Secondly, we show that upon chronic dyskinesiogenic treatment with L-DOPA, dSPNs segregate in two clusters exhibiting quite distinct morphological-physiological adaptions. The so-called cluster-1 dSPNs exhibit no sign of dendritic recovery, but a normalization of their intrinsic excitability, similar to the response observed in dSPNs in the mouse model of LID^3^. In contrast, dSPNs in the so-called cluster-2 showed an increase in dendritic branching, along with a drastic reduction in excitability. Interestingly, FosB/∆FosB expression appears to be linked with cluster-2, but not cluster-1 dSPNs.

Dendritic atrophy is a pathological process occurring in PD and several other basal ganglia disorders, such as Huntington’s disease^10–12,24,25^. Our previous work in 6-OHDA-lesioned mice has shown that dendritic atrophy occurs in both SPN subpopulations, which was confirmed in some studies, but not others^13–15^. Investigations of dSPNs and iSPNs in species other than the mouse have been limited due to the lack of reliable labeling procedures. Retrograde tracing labels specific neuronal populations based on their axonal targets. However, for SPNs it comes with the limitation that the GPe is not only innervated by iSPNs but also receives axon collaterals from dSPNs^26^. The present study was therefore limited to dSPNs, as we could only unequivocally identify this population with our approach. The FuG-B viral vectors used in this study were very efficient in retrogradely transducing dSPNs. Furthermore, our pilot experiments indicated that the extent of transduced cells in the striatum was vector titer-dependent (*data not shown*). This technique therefore presents a valuable tool for future applications, as it allows for the targeted manipulation of dSPNs to adjustable degrees.

To our knowledge, this is the first study to address morphological and electrophysiological changes of individual dSPNs in a rat model of PD and LID. Previous studies have reported increased excitability^27^ and reduced dendritic arborization^15^ in rat SPNs after 6-OHDA lesion but could not attribute these changes to iSPNs or dSPNs. We here fill this gap, at least partially, by showing that rat dSPNs are hyper-excitable and display dendritic atrophy after chronic DA-denervation, similarly to the findings obtained in the mouse.

Our observation that L-DOPA treatment triggers two distinct responses in dSPNs raises several questions. One question is whether dSPNs in cluster-1 and 2 were different even before the lesion. The choice of labeling technique and the verifications performed using BAC-*drd1a*-tdTomato mice indicate that both types of cells are classical dSPNs, projecting to the SNr and expressing the D1 receptor. However, whether or not all SPNs do indeed express only one class of DA receptors has been discussed for more than two decades^28–30^. A recent study in the mouse identified a group of SPNs expressing both D1- and D2-receptors, and suggested that these cells form a separate population with distinct somatic and dendritic morphology^13^. Interestingly, these D1/D2-positive SPNs also undergo specific adaptations in a model of PD. However, it is unlikely that either cluster-1 or cluster-2 cells corresponded to the “D1/D2 subgroup”, which amounts to less than 2% of all SPNs in the dorsolateral striatum. This low number is at odds with the finding that 50% of dSPNs segregated into either cluster-1 or 2. Additionally, it is not known where the projection targets of D1/D2-SPNs are located, and if they can be targeted with our retrograde labeling approach.

The second obvious question is how cluster-1 and cluster-2 dSPN are linked to LID. Here, several lines of argumentation have to be considered. The classical framework of LID assumes hyperactivity of the direct pathway as the primary cause of the involuntary movements. Studies using broad optogenetic^31,32^ or chemogenetic^33^ stimulation of dSPNs support this hypothesis, even though the induced abnormal involuntary movements do not reach the same severity as with L-DOPA treatment. A new hypothesis was recently brought forward by Girasole and colleagues^34^. They used targeted recombination in active populations (TRAP) to capture and manipulate neurons that are active and express Fos during LID. These FosTRAPed neurons only encompassed 20% of all striatal neurons, and were largely dSPNs. Optogenetic activation of the FosTRAPed neuronal subgroup induced dyskinetic behaviors in absence of L-DOPA, and optogenetic inhibition of the same neurons reduced the severity of LID. It is tempting to speculate that this stable subgroup of dSPNs, whose activity is causally linked with LID, corresponds to one of the dSPNs clusters described here. Due to the mutual link of Fos-activation, it seems parsimonious to assume that it would be the cells in cluster-2. Morphological and electrophysiological analysis of FosTRAPed neurons could answer this question.

One may wonder how the dramatically reduced excitability of cluster-2 cells fits together with the well-accepted notion of dSPNs being overactive during LID. At first glance this may seem counterintuitive. However, it is important to note that dSPN intrinsic excitability was here measured “off” L-DOPA. In fact, a reduced intrinsic excitability in the absence of L-DOPA may be linked to an increased firing activity of the same cells “on” L-DOPA. Thus, in models of PD, iSPNs show a reduced intrinsic excitability^3^ as part of a homeostatic response to a higher firing activity^35^. It is therefore quite possible that cluster-2 dSPNs down-regulated their intrinsic excitability in an attempt to adapt to a large increase in firing rate during the expression of LID.

Thirdly, the question arises as to what drives the different responses to L-DOPA in the two dSPN clusters. In a first attempt to answer this question, we focused our attention on FosB-like transcription factors. Although the antibody used here recognizes both full-length FosB and its splice-variant ΔFosB, it is well described that the staining observed in chronically L-DOPA-treated animals is due to ΔFosB^36,37^. ΔFosB is a nuclear transcription factor with very long half-life, mediating long-lasting adaptations in neurons^38,39^. Its striatal expression has been tightly linked to the occurrence of LID in animal models^22,23,40^ and has also been reported in a post mortem study of putaminal sections from dyskinetic PD patients^41^. With chronic L-DOPA treatment, ΔFosB accumulates in a specific subset of dSPNs^23^. Although not demonstrated in this study, a causal link between neuronal accumulation of ∆FosB and dendritic remodeling appears plausible. Indeed, ∆FosB acts as a transcriptional activator of genes involved in the regulation of cytoskeletal dynamics. One of these genes codes for cyclin-dependent kinase-5, whose activity has been linked to neurite outgrowth in cultured neurons^42^ and to cocaine-induced dendritic spine proliferation in the nucleus accumbens^43^.

To our knowledge, the present study provides the first evidence that dendritic regeneration of SPNs can occur in a PD model upon DA replacement therapy. However, how this relates to the occurrence and manifestation of LID needs to be elucidated. In animal models of PD, treatments that inhibit the striatal induction of FosB/∆FosB by L-DOPA also inhibit the development of LID^44–46^. Moreover, experimentally upregulating ∆FosB makes parkinsonian animals more susceptible to LID^47,48^. Additionally, dyskinetic behaviors induced by optogenetic stimulation of dSPNs also increase the expression of FosB/∆FosB in these neurons^32^. Taken together, these previous studies are compatible with the hypothesis that the FosB-immunoreactive dSPNs in cluster-2 are positively linked to the occurrence of abnormal involuntary movements, and that their dendritic expansion represents an unwanted adaptation from a therapeutic standpoint.

Lastly, our study contributes a timely piece of information to the emerging view of dSPNs as a heterogeneous cell population. In a recent study using single cell sequencing, Gokce and colleagues^49^ found that D1 receptor-expressing neurons in the healthy striatum split into two major populations. One of these, characterized by the expression of *Foxo1*, can be further subdivided based on additional gene expression gradients. Although it remains unknown whether transcriptionally separated SPN groups also have distinct morphological or physiological phenotypes, it seems possible that preexisting molecular differences between dSPNs underlie the segregation of responses observed after L-DOPA treatment in this animal model of PD.

## Methods

All experimental procedures were approved by the Malmo-Lund Ethical Committee on Animal Research and are in adherence with directive 2010/63/EU.

### Viral vector production

GFP lentiviral vectors were produced using transient co-transfection of transfer, envelope and packaging plasmids in Human Embryonic Kidney (HEK) 293 T cells using the calcium phosphate precipitation method as previously described^50,51^. The transfer plasmid contains the DNA sequence coding for the GFP protein under the control of the human synapsin promoter and upstream the Woodchuck Hepatitis Virus Posttranscriptional Regulatory Element (WPRE) sequence. The envelope plasmid encodes the glycoproteins FuG-B, a fusion of the extracellular and trans-membrane domains of rabies virus glycoprotein (RV-G) and the cytoplasmic domain of vesicular stomatitis virus glycoprotein (VSV-G)^17^. The functional titer was estimated as previously described^51^. Briefly, HEK cells were transduced and standard qPCR protocols were used to measure the number of WPRE copies present. The functional titer of the virus was estimated by comparison to a reference virus batch of a known titer (CMV-GFP lentiviral vector titer assessed by flow cytometry). The functional titer of the FuG-B-synapsin-GFP virus was estimated at 3.1 × 10^9^ TU/mL, where TU are transducing units.

### Animals and stereotaxic surgeries

Adult, female Sprague-Dawley rats (approximately 225 g at the beginning of the experiments) were housed under a fixed 12 h light/dark cycle with free access to water and food. To induce unilateral parkinsonism, rats were injected with 6-OHDA hydrochloride into the right median forebrain bundle as previously described^52,53^.

To retrogradely label striatonigral neurons, FuG-B-GFP lentiviral vectors were injected into the substantia nigra pars reticulata at two sites: AP −5.3, ML −1.8, DV −7.5 and AP −5.3, ML −2.7, DV −7.1, relative to bregma and dura. At each site, 1.5 μL of construct (3.1 × 10^9^ TU/mL) were injected. Pilot experiments showed that this titer gives a robust GFP-labeling of cells throughout the striatum, whereas 5- and 20-times diluted titer resulted in clustered and sparse labeling (*data not shown*).

In a subset of experiments BAC-transgenic mice expressing the fluorophore tdTomato under the *drd1a* promoter (BAC-*drd1a*-tdTomato-mice) were used. The same viral construct was injected into the substantia nigra pars reticulata (1 μL, 3.1 × 10^9^ TU/mL, AP −3.4 ML −1.4 DV −4.3, relative to bregma and dura) and animals were transcardially perfused with 4% PFA eight weeks later for immunohistochemical analysis.

### Verification of lesion and LID

Rats were screened for successful lesions 14 days post 6-OHDA injection using the cylinder test of forelimb use asymmetry. Cut-off for selection was ≤25% use of the paw contralateral to the lesion. DA denervation was furthermore confirmed by tyrosine hydroxylase (TH) immunostaining at the end of each experiment (see Fig. 1B). L-DOPA treatment started five weeks after viral vector injection, and rats received daily injections of 10 mg/kg L-DOPA in combination with 12 mg/kg benserazide for 3–4 weeks. Animals in the “PD group” received injections of vehicle (saline). Abnormal involuntary movements (AIMs; axial, limb and orolingual) were rated once a week^54^ and all animals in this study were found to develop moderate-severe LID (see Fig. 1C). We recorded and imaged one neuron per brain slice and typically one to three neurons were used from one rat (Sham: 9 rats, 13 cells; PD: 11 rats, 18 cells; LID: 17 rats, 34 cells).

### Patch-clamp recordings and 2PLSM

One day after the last injection of L-DOPA, acute sagittal slices were prepared in 275 μm thickness based on established protocols^3,18,52^. GFP-positive neurons located in the dorsolateral striatum were selected for path-clamp recordings and 2PLSM. A further distinction into striatal subareas, e.g. patch or matrix, was not done. During the entire patching and imaging procedure, slices were superfused with oxygenated aCSF, containing (in mM): 124.0 NaCl, 3.0 KCl, 1.0 CaCl_2_, 2.0 MgCl_2_, 26.0 NaHCO_3_, 1.0 NaH_2_PO_4_ and 16.66 glucose. The osmolarity was typically 300–310 mOsm/L and pH 7.4. All recordings were performed at room temperature. The internal recording solution contained (in mM): 135.0 KMeSO_4_, 5.0 KCL, 0.16 CaCl_2_, 10.0 HEPES, 2.0 Mg-ATP, 0.5 Na-GTP, 5 phosphocreatine-Tris, 5.0 phospocreatine-NA, with a pH adjusted to 7.25–7.3 and osmolarity 270–280 mOsm/L.

Patch-pipettes were pulled from borosilicate glass and had a typical resistance of 3–5 mΩ. Somatic excitability was assessed in current clamp by injecting a series of increasing current steps at the soma and measuring the resulting action potential firing. Recordings were sampled at 10–20 kHz using a Multiclamp 700B amplifier (Molecular Devices) and digitized using a Digidata 1440 (Molecular Devices). Cells were filled with Alexa 568 (50μM in the internal solution) and visualized through a 63x objective (1.0 NA, Zeiss) on a Zeiss LSM710 NLO, using a MaiTai two photon laser (780 nm). For each cell four z-stacks were acquired, encompassing the entire cell. Optical sections were spaced 1 μm apart and the x-y resolution was 0.415 × 0.415 μm^2^ per pixel. The stacks were stitched together offline and reconstructed using IMARIS (Bitplane AG).

### Immunohistochemistry and confocal imaging

After concluding the above experiments, all slices and the remaining medial sections of the striatum were fixed in 4% PFA and re-sliced on a freezing microtome into thin sections (40 μm). To assess striatal DA denervation, slices were washed, blocked and incubated with a rabbit anti-tyrosine hydroxylase antibody (1:1000, Pel-Freeze) overnight at 4 °C. A biotinylated secondary antibody (1:200, BA-1000, Vectorlabs) and the VectaStain ABC Kit (Vectorlabs) were used to reveal TH-staining using the DAB reaction.

A subset of rats was transcardially perfused with 4% PFA for immunofluorescent staining. The primary antibodies used were rabbit anti-DARPP32 (1:1000, Cell Signaling; #2302) and goat anti-FosB (1:10000, Santa Cruz, sc-48). Staining was revealed using adequate antibodies, either coupled to Cy3 or Cy5 (1:250, Vectorlabs). FuG-B-GFP fluorescence was either imaged directly, or after enhancement using a chicken-anti-GFP antibody (1:20000, ab13970, Abcam) and a Alexa488-conjugated secondary antibody (1:400, Goat anti-chicken, ThermoFisher). Confocal images were taken on a Zeiss LSM710 NLO, with the pinhole set to 1 AU. Co-expression of fluorophores was assessed on single focal-plane images. GFP and Alexa488 were excited using 488 nm (Argon laser, Lasos), red and far-red fluorophores with 543 nm and 633 nm (HeNe, Zeiss).

### Data analysis and statistics

The datasets generated during and/or analyzed during the current study are available from the corresponding author on reasonable request. Data were processed and analyzed using Microsoft Excel, Prism6 (GraphPad Software Inc.), NIH ImageJ, pClamp v.10 (Molecular Devices) and IMARIS (v.7.6.1, Bitplane). Statistical analysis was done in Prism6. Group comparisons were made using ANOVA followed by Tukey’s multiple comparison test, or two-way repeated measure ANOVA followed by Tukey’s multiple comparison test. Data are presented as box plot with median and whiskers annotating minimum and maximum values, or as mean and standard error of mean. Level of significance was set at p < 0.05.
